# Artificial Intelligence-Aided Recognition of Pathological Characteristics and Subtype Classification of Superficial Perivascular Dermatitis

**DOI:** 10.3389/fmed.2021.696305

**Published:** 2021-07-16

**Authors:** Yingqiu Bao, Jing Zhang, Qiuli Zhang, Jianmin Chang, Di Lu, Yu Fu

**Affiliations:** ^1^Department of Dermatology, Beijing Hospital, National Center of Gerontology, Beijing, China; ^2^Department of Biomedical Engineering, Tsinghua University, Beijing, China; ^3^Bodhi Lab., Beijing BeYes Technology Co. Ltd., Beijing, China

**Keywords:** superficial perivascular dermatitis, skin histopathology images, multitask deep learning, pathological characteristics, subtype classification

## Abstract

**Background:** Superficial perivascular dermatitis, an important type of inflammatory dermatosis, comprises various skin diseases, which are difficult to distinguish by clinical manifestations and need pathological imaging observation. Coupled with its complex pathological characteristics, the subtype classification depends to a great extent on dermatopathologists. There is an urgent need to develop an efficient approach to recognize the pathological characteristics and classify the subtypes of superficial perivascular dermatitis.

**Methods:** 3,954 pathological images (4 × and 10 ×) of three subtypes—psoriasiform, spongiotic and interface—of superficial perivascular dermatitis were captured from 327 cases diagnosed both clinically and pathologically. The control group comprised 1,337 pathological images of 85 normal skin tissue slides taken from the edge of benign epidermal cysts. First, senior dermatologists and dermatopathologists followed the structure–pattern analysis method to label the pathological characteristics that significantly contribute to classifying different subtypes on 4 × and 10 × images. A cascaded deep learning algorithm framework was then proposed to establish pixel-level pathological characteristics' masks and classify the subtypes by supervised learning.

**Results:** 13 different pathological characteristics were recognized, and the accuracy of subtype classification was 85.24%. In contrast, the accuracy of the subtype classification model without recognition was 71.35%.

**Conclusion:** Our cascaded deep learning model used small samples to deliver efficient recognition of pathological characteristics and subtype classification simultaneously. Moreover, the proposed method could be applied to both microscopic images and digital scanned images.

## Introduction

Inflammatory dermatoses constitute a major category of skin diseases, including many common conditions such as psoriasis, lichen planus, and eczema. For cases with atypical clinical manifestation, a pathological imaging observation can distinguish inflammation subtypes based on pathological characteristics and thus provide key clues for dermatologists to formulate diagnoses and treatments. Professor A. Bernard Ackerman first proposed the structure–pattern analysis method in the 1960s, which laid the foundation for dermatopathology (1). Among the inflammatory dermatoses, superficial perivascular dermatitis is an important pathological type with four subtypes: simplex, interface, psoriasiform and spongiotic (2). However, identification of the pathological characteristics and subtypes of inflammation remains time consuming and relies on professional experience. Furthermore, the lack of senior dermatopathologists in China makes the task more difficult. Therefore, developing an efficient approach to recognize pathological characteristics and classify subtypes of inflammation is essential.

Recently, some strategies based on artificial intelligence (AI) and machine learning, including deep learning networks, have been developed and are being widely used in dermatopathology. Hekler et al. (3) established a deep learning classification model using 695 whole-slide images to distinguish melanoma or benign nevus, achieving an accuracy of 68%. In comparison, the average accuracy of 11 other dermatologists was 59.2%. Jiang et al. (4) established a deep learning segmentation model, based on 8,046 pathological images collected using a smartphone and achieved automatic recognition and segmentation of basal cell carcinoma. The model reached an intersection over union (IoU) of 0.863, a specificity of 0.969, and a sensitivity of 0.939, respectively, which were significantly better than the previous IoU (0.764) (5). specificity (0.927), and sensitivity (0.869) (6). The above studies show that deep learning models can be used to classify pathological images and recognize pathological characteristics.

Current studies are focused on skin tumors, and there has been no related study on artificial intelligence-aided recognition of pathological characteristics and classification of skin inflammation. In this study, following the structure–pattern analysis method, a cascaded deep learning framework was established in combination with a pixel-level segmentation model to achieve pathological characteristics' masks and a model to classify the subtypes of superficial perivascular dermatitis. It would be an efficient approach to recognize pathological characteristics and classify subtypes of superficial perivascular dermatitis simultaneously.

The incidence of inflammatory dermatoses, including many common conditions such as psoriasis, eczema, and lichen planus, is high in China. Because some inflammatory dermatoses with atypical manifestations are difficult to distinguish, histopathology images play an important role in helping clinicians make the right decision. Ackerman's structure–pattern analysis is a useful means for identifying the pathological subtypes of inflammatory skin diseases (7), and superficial perivascular dermatitis is the most common inflammatory skin disease. However, given the complex pathological patterns of inflammatory skin diseases and the lack of senior dermatopathologists, pathological identification remains time consuming and depends on professional experience. In recent years, the progress of AI technology in the medical field is particularly reflected in the rapid recognition and classification of various medical images. Computer vision and deep learning methods are being increasingly applied in dermatology and dermatopathology, and dermatologists are generally positive about the increased use of AI. Among 718 physicians from 91 countries, 72.3% agreed or strongly agreed that AI would improve dermatopathology (8). In 2017, a Stanford team used deep learning to automatically classify nearly 130,000 dermoscopic images of clinical skin lesions (9). They employed the “GoogleNet Inception-V3” classification framework, used transfer learning to fine-tune model parameters, and obtained the final convolutional neural network model. Using the model, the team completed the diagnostic tasks of melanoma and benign pigmented moles and achieved a recognition result equivalent to that of a dermatologist. Olsen et al. (10) established a deep learning classification model using 450 whole-slide images to classify basal cell carcinoma, dermal nevus, and seborrheic keratosis, obtaining the area under the curve results of 0.99, 0.97, and 0.99, respectively. Achi et al. (11) established a deep learning classification model using 128 whole-slide images and achieved an image-based accuracy of 95% and a test set-based accuracy of 100% in classifying four types of lymphomas: benign lymph node, diffuse large B-cell lymphoma, Burkitt lymphoma, and small lymphocytic lymphoma. Current studies primarily focus on recognition of dermoscopic images and pathological recognition of benign/malignant lesions. There was no relevant research on the recognition of pathological characteristics and classification of skin inflammation (12). This study was the first to use AI technology to assist in pathological recognition and classification of inflammatory dermatosis.

The challenges of recognizing mainly focus on low contrast and having cluttering background in the process of pathological characteristics' recognition. Moreover, the whole-slide is high resolution. To solve the above, this study uses pipeline processing for pathological characteristics' recognition and subtype classification. First, the pathological characteristics are recognized by segmentaion model at 4 × and 10 × separately. The pathological characteristics are typically recognizable under the current magnification and contribute to the subtype classification, so as to reduce the influence of low contrast and cluttering background as much as possible. In addition, the 4 × images and 10 × images belonging to the same slide are used as a set to simulate the high-resolution mode of the whole-slide for classification. For the model selection, the strategy is to use better-performing segmentation algorithms and classification algorithms. Finally we choose deeplabV3+ from deeplabV1, V2, V3, V3+, which is more sensitive to different scales, and EfficientNet-B1 which can carry out multi-channel input.

## Materials and Methods

### Dataset

This was a retrospective study and the samples were obtained from the pathological slides that have been preserved at the Dermatology Department of Beijing Hospital from Jan 2015 to Jun 2020. Senior dermatologists and dermal pathologists selected pathological slides that had been diagnosed both clinically and pathologically, and the most representative diseases of the three subtypes of superficial perivascular dermatitis—psoriasiform, spongiotic and interface—were chosen, including psoriasis, acute eczema and contact dermatitis, lichen planus, and lichen sclerosus et atrophicus. In total, the histopathology slides of 412 cases were selected to collect 3,954 images from 327 cases of three subtypes and 1,337 images from 85 cases of normal skin histopathology slides taken from the edge of benign epidermal cysts. There were 5,291 images in total, including 4 × images and 10 × images. The details of the dataset are shown in Table 1. One subtype classification sample set contained two 4 × images and two 10 × images of the same slide. In addition, 2,435 images were captured from microscope screenshots and the other 2,856 images were captured from digitally scanned whole-slide images.

**Table 1:** The details of superficial perivascular dermatitis dataset.

**Magnifications**	**Psoriasiform**	**Spongiotic**	**Interface**	**Normal**	**Total**
	**Train/Test images**	**Train/Test Images**	**Train/Test images**	**Train/Test images**	**Train/Test images**
4 ×	564/44	507/38	396/50	507/44	1974/176
10 ×	772/58	747/73	592/113	714/72	2825/316

All images were labeled manually by senior dermatologists and dermatopathologists. This study was approved by the Ethics Committee of Beijing hospital.

### Labeling of Pathological Characteristics

**Figure 1:**
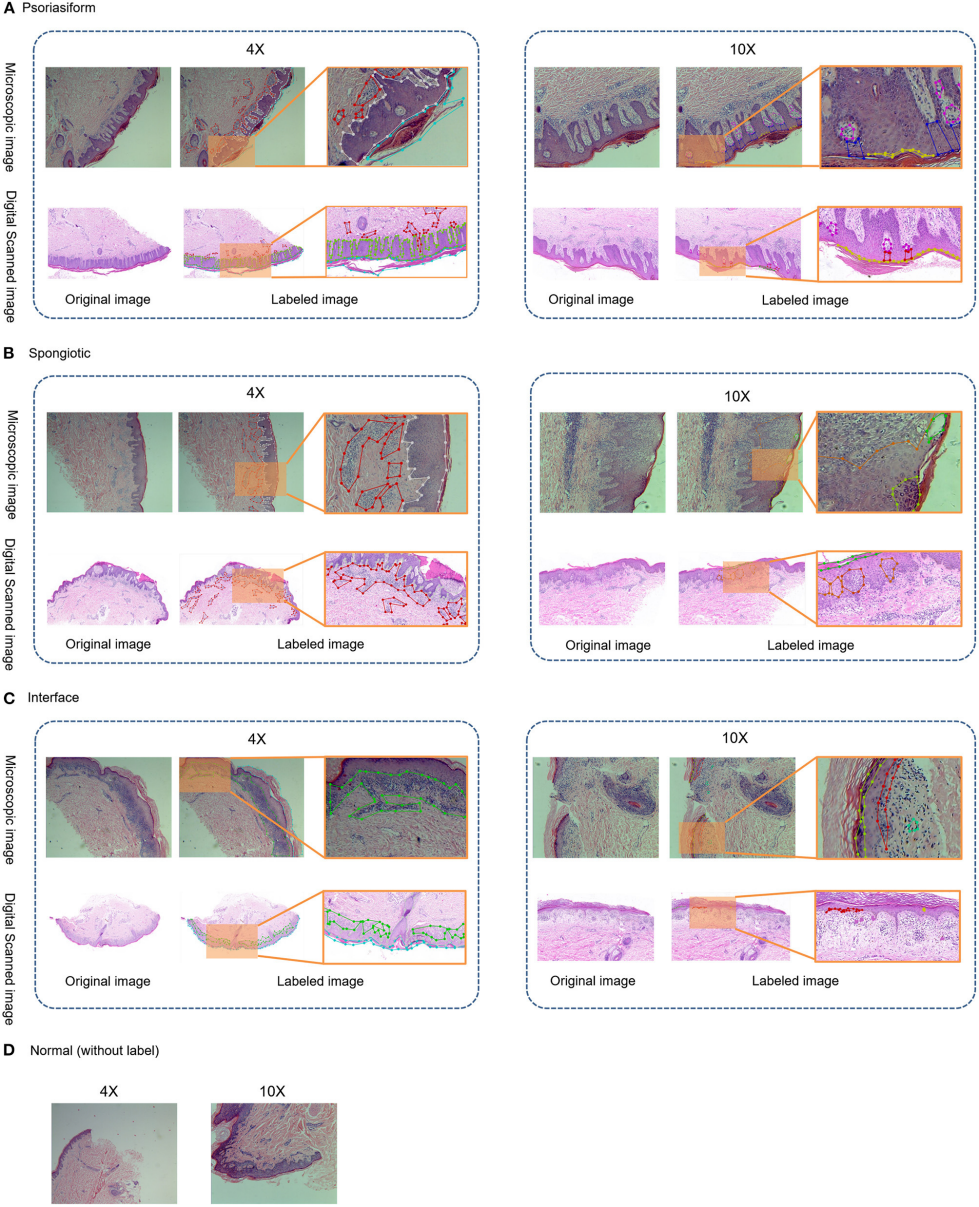
Original and labeled images of different subtypes of superficial perivascular dermatitis. (Note: The labels refer to Table 2 and the generated annotation information of the labeled image is in the json file).

The principle of structure–pattern analysis method is observing the pathological characteristics ranging from low magnification to high magnification and then identifying the subtypes of superficial perivascular dermatitis, which was based on the labeling of pathological characteristics in this study. Rather than labeling all pathological changes, the pathological characteristics were labeled by considering their contribution to subtype classification, which could help to reduce the deviation in identification between physicians. There are two principles for selecting pathological characteristics in this study. One is that the characteristic is clearly visible at the current magnification, and then it is judged by experienced dermatopathologists that this characteristic has a significant contribution to the subtype of inflammation. For example, hyperkeratosis, acanthosis and infiltration of perivascular inflammatory cells are more obvious at 4 × than 10 ×, which are the typical characteristics of psoriasiform subtype at 4 ×. The labeling regions of the three subtypes are shown in Figure 1 and Table 2. The labeling software was labelme.exe compiled by Beyes Tech.

**Table 2:** Labels and labeling magnifications.

**Magnifications**	**Psoriasiform**	**Interface**	**Spongiotic**
4 ×	Hyperkeratosis, acanthosis, infiltration of perivascular inflammatory cells	Hyperkeratosis, acanthosis, lichenoid infiltration of inflammatory cells	Hyperkeratosis, acanthosis, infiltration of perivascular inflammatory cells
10 ×	Parakeratosis, hypogranulosis, angiectasis of dermal papillae and thinning of the suprapapillary epidermis	Melanophage, hypergranulosis, liquefaction degeneration of basal cells	Parakeratosis, spongiosis, hypergranulosis, blister

### Cascaded Deep Learning Framework

**Figure 2:**
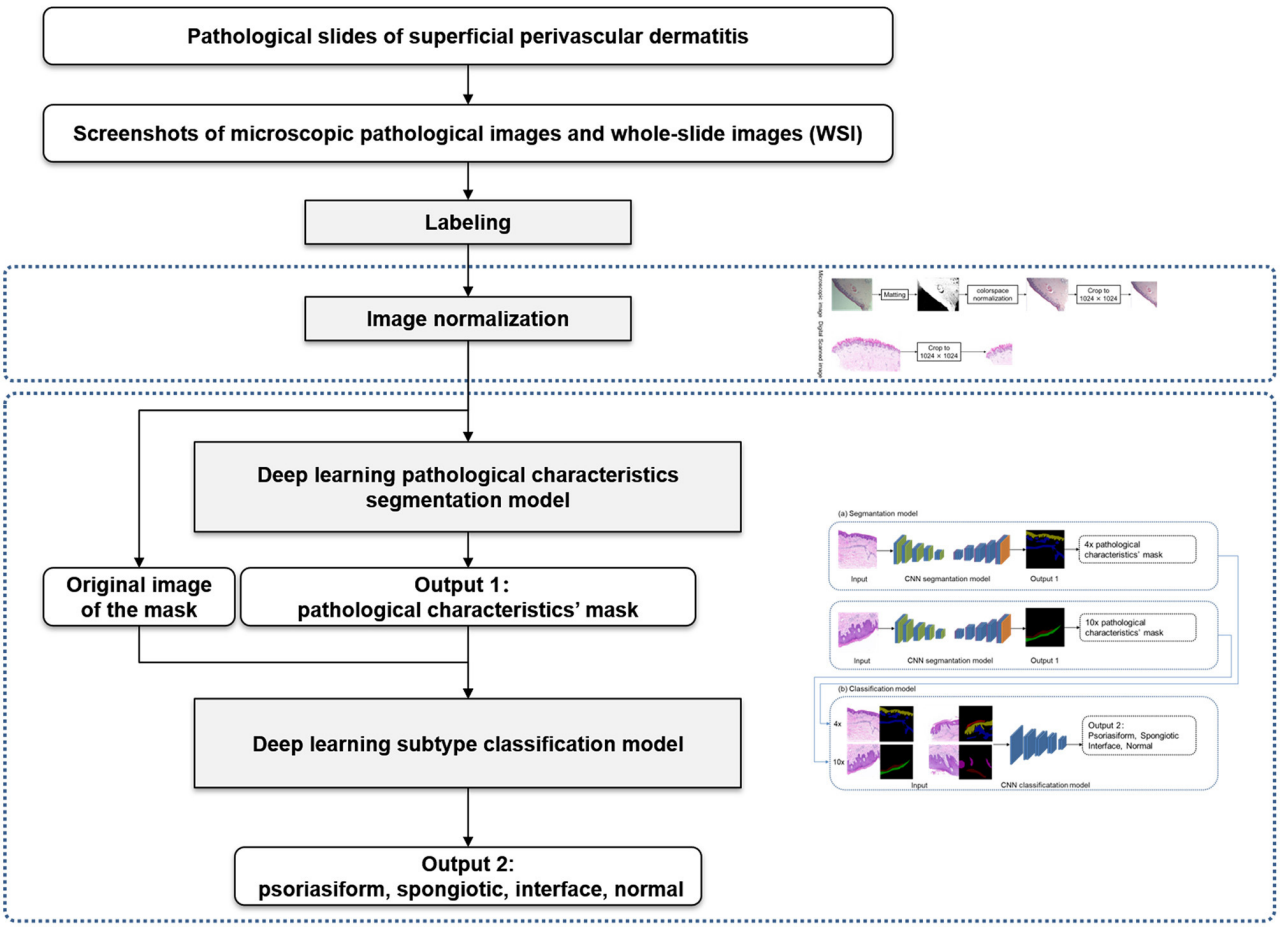
Overall framework of the model.

The overall framework of this model was composed of preprocessing and cascaded recognition model for pathological characteristics and subtype classification, shown in Figure 2. After labeling, each sample was normalized (preprocessing) and treated using the deep learning models for segmentation and classification, thus obtaining its pathological characteristics' mask and subtype of superficial perivascular dermatitis.

### Normalization (Preprocessing)

**Figure 3:**
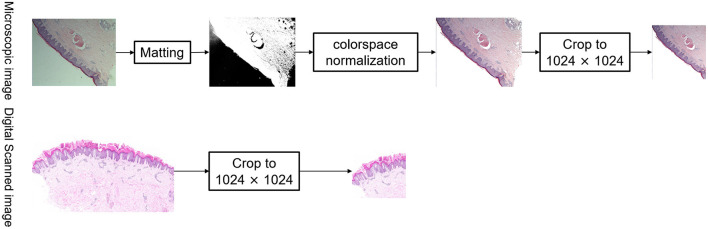
Normalization of images.

Microscopic images were normalized *via* matting of the foreground and background, color space normalization, and cropping of digitally scanned images to 1,024 × 1,024, as shown in Figure 3. In order to unify the color of the microscope images to the digital scanned images, the algorithm used in this study is background matting, which is the trimap-free automatic matting algorithm that utilizes a casually captured background (13). Thus, microscopic images and digitally scanned images were made consistent in color space and size to reduce differences and facilitate uniform recognition of pathological characteristics and subtype classification modeling.

### Modeling of Recognition of Pathological Characteristics

The sampled 4 × and 10 × images were used for recognition of pathological characteristics. The deep learning segmentation model “deeplabV3+” was used for supervised learning. The region masks generated by 4 × images were hyperkeratosis, acanthosis, inflammatory cell infiltration, and lichenoid infiltration. The region masks generated by 10 × images were parakeratosis, spongiosis, melanophages, hypergranulosis, hypogranulosis, blister, liquefaction degeneration of basal cells, angiectasis of dermal papillae, and thinning of the suprapapillary epidermis. The DeepLabV3+ parameter settings of training: LR = 0.01, LR_GAMMA = 0.1, BATCHES = 4,EPOCHS = 500.

### Modeling of Subtype Classification

**Figure 4:**
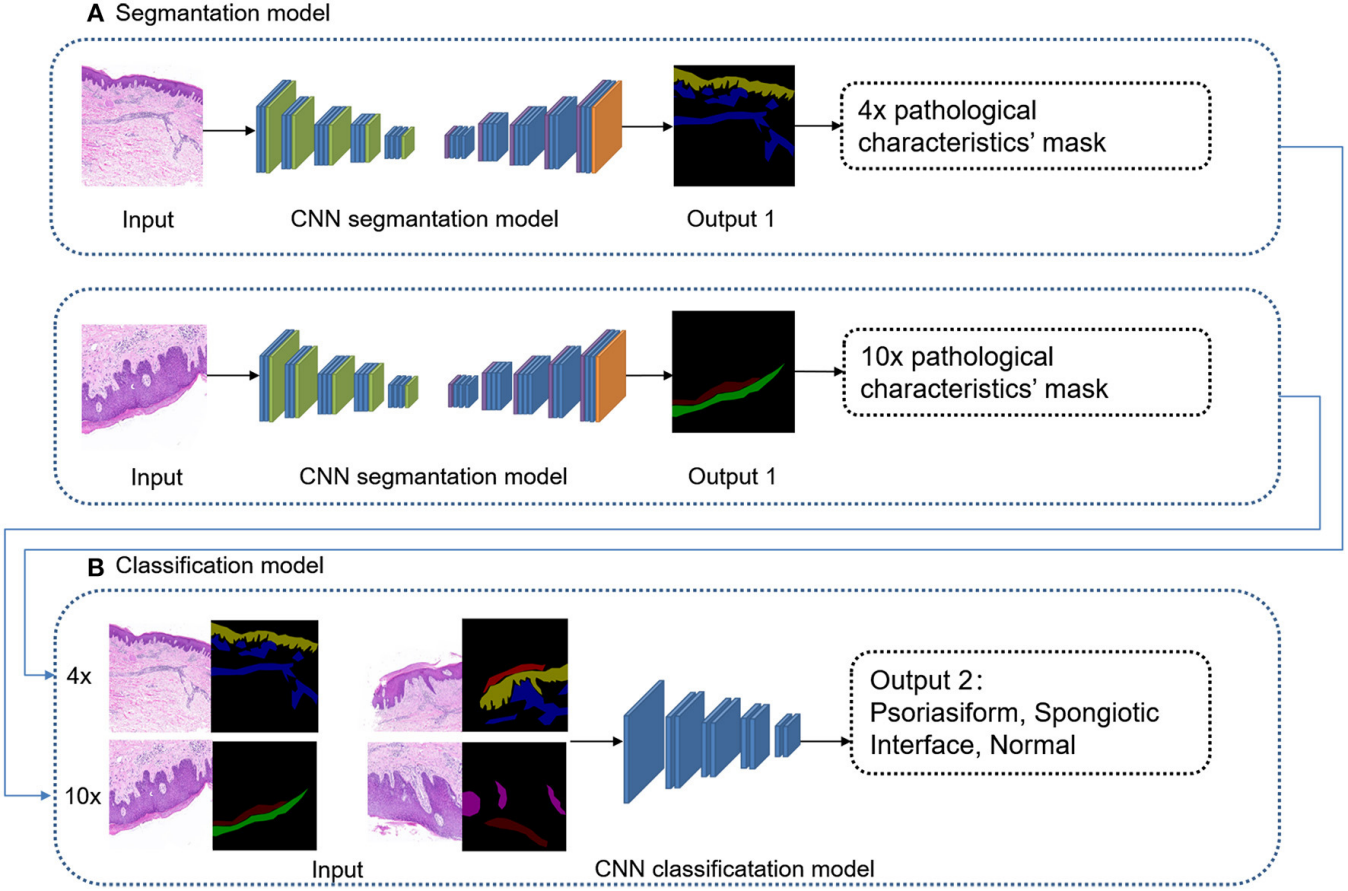
Cascaded deep learning model of cascaded pathological characteristics recognition and subtype classification.

The deep learning classification model “EfficientNet-B1” was used for supervised learning. The input was 4 images' channles, followed by 4 × original image and its pathological characteristics' mask, 10 × original image and its pathological characteristics' mask. The output was four subtypes: psoriasiform, spongiotic, interface, and normal, as shown in Figure 4. The original images were randomly increased with Gaussian noise and salt-and-pepper noise to avoid overfitting of the training set. The adding method was 45% Gaussian noise and 45% salt-and-pepper noise. The Efficientnet-B1 parameter settings of training: LR = 1e-4, batch_size = 8, EPOCH = 201, betas = (0.9, 0.999), eps = 1e-9.

Finally, the performance of our model is evaluated by accuracy, sensitivity, and specificity. The formula for sensitivity and specificity is:

$$\begin{array}{l} sensitivity=TP/P \end{array}$$

TP, The number of sets that are actually positive and classified as positive; P, The number of the sets that are actually positive.

$$\begin{array}{l} specificity=TN/N \end{array}$$

TN, The number of sets that are actually negative and classified as negative; N, The number of the sets that are actually negative.

## Results

### Modeling Results and Evaluation

**Figure 5:**
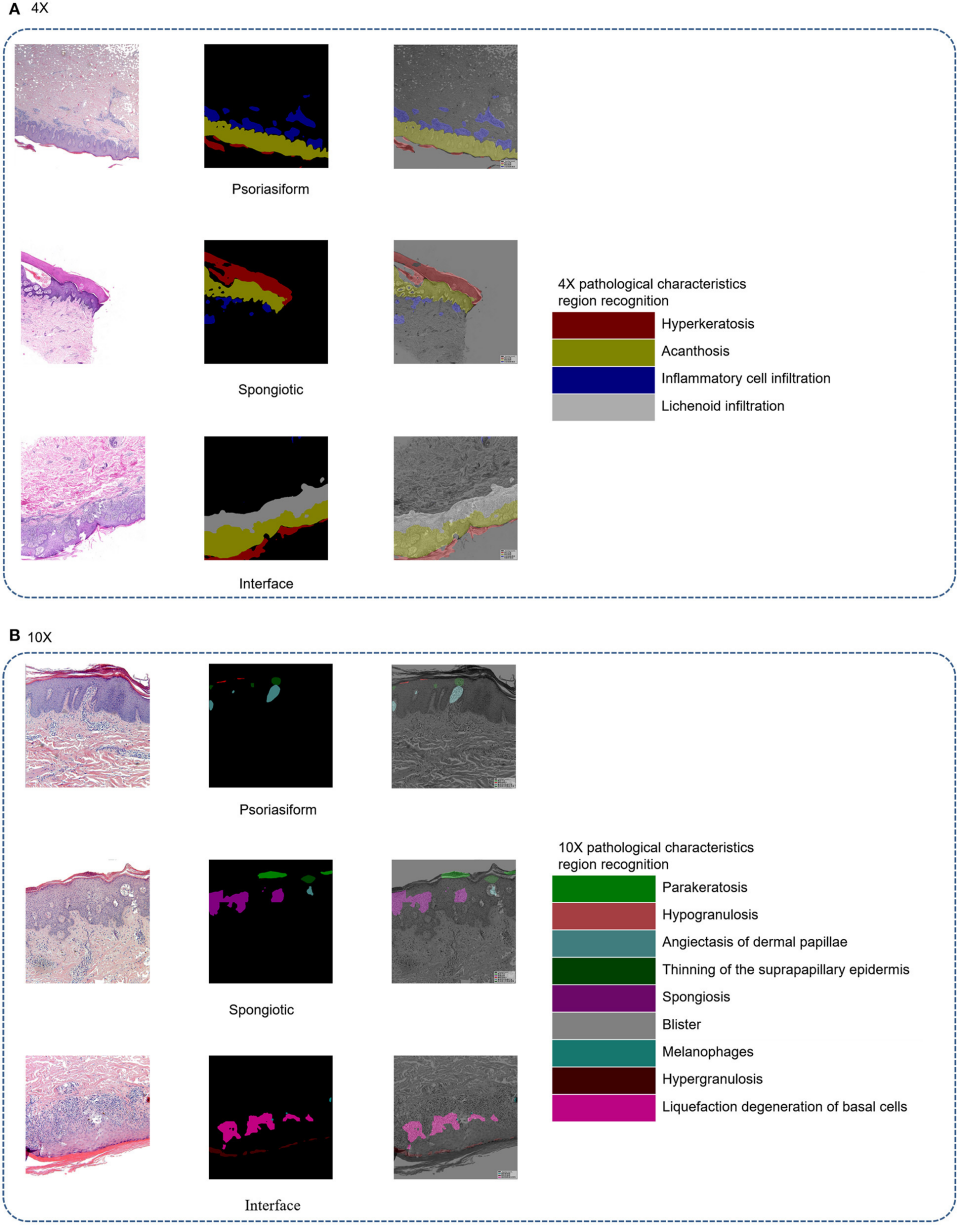
Results of pathological characteristics region recognition.

Our subtype classification accuracy of the four types in the test set was 85.24%, and the sensitivity and the specificity were 67.46 and 89.09%, respectively. In contrast, the accuracy of Efficientnet-B1 subtype classification without recognition was 71.35%; the accuracy of Efficientnet-B1 cascaded U-Net was 79.88%; the accuracy of ResNet152 cascaded was 80.76%. Table 3 shows the subtype classification results, and Table 4 shows the different model parameter settings of training. The accuracy of each subtype of our model is 99.79%(normal), 81.12%(pongiotic), 83.69%(interface), and 64.58%(psoriasiform). Therefore, the cascaded recognition model for pathological characteristics was considerably effective in the subtype classification. Meanwhile, for the 4 × images, the highest DICE coefficient was 0.8336 (acanthosis). For the 10 × images, the highest DICE coefficient was 0.7262 (hypergranulosis). Table 5 and Figure 5 show the recognition results.

**Table 3:** Results of subtype classification of superficial perivascular dermatitis.

**Classification model**	**Classification accuracy (%)**
Efficientnet-B1 without recognition	71.35
Efficientnet-B1 cascaded U-Net	79.88
ResNet152 cascaded deeplab V3+	80.76
Our model (Efficientnet-B1 cascaded deeplab V3+)	85.24

**Table 4:** The different model parameter settings of training.

**Type of model**	**Model name**	**Parameter settings of training**
The model of recognition of pathological characteristics	Efficientnet-B1	LR =1e-4 batch_size = 8 EPOCH = 201 Betas = (0.9, 0.999) eps = 1e-9
	U-Net	LR = 0.001 batch_size = 16 epochs = 100 img_scale = 0.5
The model of subtype classification	deeplab V3+	LR = 0.01 LR_GAMMA = 0.1 BATCHES = 4 EPOCHS = 500
	ResNet152	learning_rate = 0.01 batch_size = 4 epoch =500

**Table 5:** Results of pathological characteristics recognition.

**Magnifications**	**Pathological characteristics region**	**DICE coefficient**
4 ×	Hyperkeratosis	0.6742
	Acanthosis	0.8336
	Inflammatory cell infiltration	0.5816
	Lichenoid infiltration	0.7729
10 ×	Parakeratosis	0.1684
	Spongiosis	0.3003
	Melanophages	0.0121
	Hypergranulosis	0.7262
	Hypogranulosis	0.1621
	Blister	0.1491
	Liquefaction degeneration of basal cells	0.2239
	Angiectasis of dermal papillae	0.4246
	Thinning of the suprapapillary epidermis	0.4076

## Discussion

The principle of structure–pattern analysis is observing the pathological characteristics from low magnification to high magnification and identifying the subtypes of skin inflammations to provide key clues for a specific diagnosis. On the basis of this, a cascaded deep learning algorithm framework was proposed with cascaded pathological characteristics recognition and subtype classification. The pixel-by-pixel segmentation deep learning model “deeplabV3+” was employed to recognize pathological characteristics. Regions that contribute considerably to a diagnosis under a 4 × magnification and 10 × magnification were extracted through supervised learning for obtaining the pathological characteristics' masks of the original images and achieve recognition results with high consistency. The deep learning model “EfficientNet-b1” was employed for subtype classification. The mask output from the recognition model for pathological characteristics was combined with an image of the same section into a set of inputs, which jointly perform supervised learning to deliver accurate subtype classification.

In this study, 13 different pathological characteristics were recognized which have a significant contribution to the subtype of inflammation. For dermatopathologists, identifying these pathological characteristics under different microscope multiples also brings important clues for the final diagnosis of the disease. The DICE coefficient ranges from 0 to 1 and the higher the DICE coefficient means the better the recognition effect. Thus, the acanthosis(DICE coefficient, 0.8,336), lichenoid infiltration(DICE coefficient, 0.7,729), hypergranulosis(DICE coefficient, 0.7,262), hyperkeratosis(DICE coefficient, 0.6,742), were better recognized. These high recognition regions can make classification more accurate. As a result, lichenoid infiltration is the interface unique pathological characteristic, and its high recognition made the classification accuracy of interface subtype higher than psoriasiform and spongiotic. Altogether, the accuracy of subtype classification is 85.24%, and the sensitivity and the specificity to be 67.46 and 89.09%. This result is very close to the actual diagnostic accuracy of dermatopathologists in clinical work. As far as we know, the types of inflammatory skin diseases are complex. It is difficult to be specific dermatoses only by pathological diagnoses. The classification of subtypes and differential diagnosis given by pathologists according to the pathological characteristics have been the greatest help for clinicians. Our results have greatly reduced the artificial subjectivity of dermatopathologists. Compared with the previous recognition of benign or malignant tumors (14), the pathological characteristics involved in inflammation are dominated by cell infiltration and tissue structure changes, which involve multiple types of cells significantly complicated. In this study, the advantages of the pixel-level region segmentation model and the deep learning classification model were fully integrated, coupled with recognition of pathological characteristics, to render the subtype classification results more explanatory. The accuracy of subtype classification without recognition model was only 71.35%, indicating that the performance of the cascaded deep learning model was considerably improved by the recognition of pathological characteristics.

This study was the first to focus on AI-aided pathological recognition of skin inflammation. Using fine-layered image labeling of small sample size, the structure–pattern analysis principle was simulated to simultaneously achieve automatic recognition of pathological characteristics and subtype classification. The color spaces of microscopic images were normalized into digitally scanned images. Both categories of images were recognizable. The three subtypes of inflammatory dermatoses selected in this study had diverse different pathological characteristics compared with normal tissues. Therefore, simplex subtype was not included because of the few pathological characteristics. As this study is the basic framework, the sample size in the future will be expanded to cover simplex subtype and the cascaded model will be generalized.

Although AI has great potential in aiding the recognition of pathological patterns of inflammatory dermatoses, the final diagnosis depends on the experience of the dermatopathologists and dermatologists. Our work would provide a valuable approach in allowing AI to facilitate dermatopathology better in the future.

## Data Availability Statement

The raw data supporting the conclusions of this article will be made available by the authors, without undue reservation.

## Author Contributions

YB and JZ carried out experiments and analyzed data. YF and JC conceived experiments and analyzed data. QZ and DL analyzed data. All the authors were involved in writing the paper and had final approval of the submitted and published versions.

## Conflict of Interest

JZ and DL were employed by company Beijing BeYes Technology Co. Ltd. The remaining authors declare that the research was conducted in the absence of any commercial or financial relationships that could be construed as a potential conflict of interest.
