# Supplementary material for: Artificial Intelligence-Aided Recognition of Pathological Characteristics and Subtype Classification of Superficial Perivascular Dermatitis
Source: Front Med (Lausanne). 2021 Jul 16;8:696305. doi: 10.3389/fmed.2021.696305 (PMC8322609; doi:10.3389/fmed.2021.696305)
Supplement: Supplementary file 1 [file Data_Sheet_1.pdf]

```python
# ----------------------------------------
# Matting for data pre- is use for the microscopic images
# ----------------------------------------
import numpy as np
import torch
import torch.optim as optim
import torch.nn as nn
from torch.autograd import Variable
from torch.nn import functional as F
import argparse
import time
import re
import os
import sys
import bdcn
from datasets.dataset import Data
import cfg
import log
import cv2

def adjust_learning_rate(optimizer, steps, step_size, gamma=0.1, logger=None):
    """Sets the learning rate to the initial LR decayed by 10 every 30 epochs"""

    for param_group in optimizer.param_groups:
        param_group['lr'] = param_group['lr'] * gamma
        if logger:
            logger.info('%s: %s' % (param_group['name'], param_group['lr']))


def cross_entropy_loss2d(inputs, targets, cuda=False, balance=1.1):
    """
    :param inputs: inputs is a 4 dimensional data nx1xhxw
    :param targets: targets is a 3 dimensional data nx1xhxw
    :return:
    """
    n, c, h, w = inputs.size()
    weights = np.zeros((n, c, h, w))
    for i in range(n):
        t = targets[i, :, :, :].cpu().data.numpy()
        pos = (t == 1).sum()
        neg = (t == 0).sum()
        valid = neg + pos
        weights[i, t == 1] = neg * 1. / valid
        weights[i, t == 0] = pos * balance / valid
    weights = torch.Tensor(weights)
    if cuda:
        weights = weights.cuda()
    inputs = F.sigmoid(inputs)
    loss = nn.BCELoss(weights, size_average=False)(inputs, targets)
    return loss

def train(model, args):
    data_root = cfg.config[args.dataset]['data_root']
    print(data_root)
    data_lst = cfg.config[args.dataset]['data_lst']
    if 'Multicue' in args.dataset:
        data_lst = data_lst % args.k
    mean_bgr = np.array(cfg.config[args.dataset]['mean_bgr'])
    yita = args.yita if args.yita else cfg.config[args.dataset]['yita']
    crop_size = args.crop_size
    train_img = Data(data_root, data_lst, yita, mean_bgr=mean_bgr,
    crop_size=crop_size)
    trainloader = torch.utils.data.DataLoader(train_img,
        batch_size=args.batch_size, shuffle=True, num_workers=5)

    params_dict = dict(model.named_parameters())
    base_lr = args.base_lr
    weight_decay = args.weight_decay
    logger = args.logger
    params = []
    for key, v in params_dict.items():
```

```
    if re.match(r'conv[1-5]_[1-3]_down', key):
        if 'weight' in key:
            params += [{'params': v, 'lr': base_lr*0.1, 'weight_decay':
            weight_decay*1, 'name': key}]
        elif 'bias' in key:
            params += [{'params': v, 'lr': base_lr*0.2, 'weight_decay':
            weight_decay*0, 'name': key}]
    elif re.match(r'.*conv[1-4]_[1-3]', key):
        if 'weight' in key:
            params += [{'params': v, 'lr': base_lr*1, 'weight_decay':
            weight_decay*1, 'name': key}]
        elif 'bias' in key:
            params += [{'params': v, 'lr': base_lr*2, 'weight_decay':
            weight_decay*0, 'name': key}]
    elif re.match(r'.*conv5_[1-3]', key):
        if 'weight' in key:
            params += [{'params': v, 'lr': base_lr*100, 'weight_decay':
            weight_decay*1, 'name': key}]
        elif 'bias' in key:
            params += [{'params': v, 'lr': base_lr*200, 'weight_decay':
            weight_decay*0, 'name': key}]
    elif re.match(r'score_dsn[1-5]', key):
        if 'weight' in key:
            params += [{'params': v, 'lr': base_lr*0.01, 'weight_decay':
            weight_decay*1, 'name': key}]
        elif 'bias' in key:
            params += [{'params': v, 'lr': base_lr*0.02, 'weight_decay':
            weight_decay*0, 'name': key}]
    elif re.match(r'upsample_[248](_5)?', key):
        if 'weight' in key:
            params += [{'params': v, 'lr': base_lr*0, 'weight_decay':
            weight_decay*0, 'name': key}]
        elif 'bias' in key:
            params += [{'params': v, 'lr': base_lr*0, 'weight_decay':
            weight_decay*0, 'name': key}]
    elif re.match(r'.*msblock[1-5]_[1-3]\.conv', key):
        if 'weight' in key:
            params += [{'params': v, 'lr': base_lr*1, 'weight_decay':
            weight_decay*1, 'name': key}]
        elif 'bias' in key:
            params += [{'params': v, 'lr': base_lr*2, 'weight_decay':
            weight_decay*0, 'name': key}]
    else:
        if 'weight' in key:
            params += [{'params': v, 'lr': base_lr*0.001, 'weight_decay':
            weight_decay*1, 'name': key}]
        elif 'bias' in key:
            params += [{'params': v, 'lr': base_lr*0.002, 'weight_decay':
            weight_decay*0, 'name': key}]
# optimizer = torch.optim.SGD(params, momentum=args.momentum,
#     lr=args.base_lr, weight_decay=args.weight_decay)
optimizer = torch.optim.Adam(params,
    lr=args.base_lr, weight_decay=args.weight_decay)
start_step = 1
mean_loss = []
cur = 0
pos = 0
data_iter = iter(trainloader)
iter_per_epoch = len(trainloader)
logger.info('*'*40)
logger.info('train images in all are %d ' % iter_per_epoch)
logger.info('*'*40)
for param_group in optimizer.param_groups:
    if logger:
        logger.info('%s: %s' % (param_group['name'], param_group['lr']))
start_time = time.time()
if args.cuda:
    model.cuda()
if args.resume:
    logger.info('resume from %s' % args.resume)
    state = torch.load(args.resume)
```

```
        start_step = state['step']
        optimizer.load_state_dict(state['solver'])
        model.load_state_dict(state['param'])
    model.train()
    batch_size = args.iter_size * args.batch_size
    for step in range(start_step, args.max_iter + 1):
        optimizer.zero_grad()
        batch_loss = 0

        # import cv2

        for i in range(args.iter_size):
            if cur == iter_per_epoch:
                cur = 0
                data_iter = iter(trainloader)
            images, labels = next(data_iter)
            # print(images.shape,labels)
            #
            cv2.imshow("image",images.numpy()[0].transpose((2,1,0)).astype(np.uint8))
            #
            cv2.imshow("label",(labels.numpy()[0].transpose((2,1,0))*255).astype(np.ui
            nt8))
            # cv2.waitKey(0)
            # exit()
            if args.cuda:
                images, labels = images.cuda(), labels.cuda()
            images, labels = Variable(images), Variable(labels)
            out = model(images)
            # out2 = [F.sigmoid(out[-1]).cpu().data.numpy()[0, 0, :, :]]
            # print("out2",out2)
            # print("labels",labels)
            loss = 0
            # for k in range(10):
            #     loss += args.side_weight*cross_entropy_loss2d(out[k], labels,
            args.cuda, args.balance)/batch_size

            side_weight_list = [0.2,0.4,0.6,0.8,1.0,1.0,0.8,0.6,0.4,0.2]
            for k in range(10):
                loss +=
                side_weight_list[k]*args.side_weight*cross_entropy_loss2d(out[k],
                labels, args.cuda, args.balance)/batch_size
            loss += args.fuse_weight*cross_entropy_loss2d(out[-1], labels,
            args.cuda, args.balance)/batch_size
            loss.backward()
            # batch_loss += loss.data[0]
            batch_loss += loss.item()  #mymodify
            cur += 1
        # update parameter
        optimizer.step()
        if len(mean_loss) < args.average_loss:
            mean_loss.append(batch_loss)
        else:
            mean_loss[pos] = batch_loss
            pos = (pos + 1) % args.average_loss
        if step % args.step_size == 0:
            adjust_learning_rate(optimizer, step, args.step_size, args.gamma)
        if step % args.snapshots == 0:
            torch.save(model.state_dict(), '%s/bdcn_%d_rate%d.pth' %
            (args.param_dir, step ,args.rate))
            state = {'step':
            step+1,'param':model.state_dict(),'solver':optimizer.state_dict()}
            torch.save(state, '%s/bdcn_%d_rate%d.pth.tar' % (args.param_dir, step
            ,args.rate))
        if step % args.display == 0:
            tm = time.time() - start_time
            logger.info('iter: %d, lr: %e, loss: %f, time using: %f(%fs/iter)' %
            (step,
                optimizer.param_groups[0]['lr'], np.mean(mean_loss), tm,
                tm/args.display))
            start_time = time.time()
```

```python
def main():
    args = parse_args()
    logger = log.get_logger(args.log)
    args.logger = logger
    logger.info('*'*80)
    logger.info('the args are the below')
    logger.info('*'*80)
    for x in args.__dict__:
        logger.info(x+','+str(args.__dict__[x]))
    logger.info(cfg.config[args.dataset])
    logger.info('*'*80)
    os.environ['CUDA_VISIBLE_DEVICES'] = args.gpu
    if not os.path.exists(args.param_dir):
        os.mkdir(args.param_dir)
    # torch.manual_seed(long(time.time()))
    #my modify
    torch.manual_seed(int(time.time()))
    model = bdcn.BDCN(pretrain=args.pretrain, logger=logger,rate2=args.rate)
    if args.complete_pretrain:
        model.load_state_dict(torch.load(args.complete_pretrain))
    logger.info(model)
    train(model, args)

def parse_args():
    parser = argparse.ArgumentParser(description='Train BDCN for different args')
    parser.add_argument('-d', '--dataset', type=str, choices=cfg.config.keys(),
        default='bsds500', help='The dataset to train')
    parser.add_argument('--param-dir', type=str, default='params',
        help='the directory to store the params')
    parser.add_argument('--lr', dest='base_lr', type=float, default=1e-4,
        help='the base learning rate of model')
    parser.add_argument('-m', '--momentum', type=float, default=0.9,
        help='the momentum')
    parser.add_argument('-c', '--cuda', action='store_true',default=True,
        help='whether use gpu to train network')
    parser.add_argument('-g', '--gpu', type=str, default='0',
        help='the gpu id to train net')
    parser.add_argument('--weight-decay', type=float, default=0.0002,
        help='the weight_decay of net')
    parser.add_argument('-r', '--resume', type=str, default=None,
        help='whether resume from some, default is None')
    parser.add_argument('-p', '--pretrain', type=str, default=None,
        help='init net from pretrained model default is None')
    parser.add_argument('--max-iter', type=int, default=6000,
        help='max iters to train network, default is 40000')
    parser.add_argument('--iter-size', type=int, default=10,
        help='iter size equal to the batch size, default 10')
    parser.add_argument('--average-loss', type=int, default=50,
        help='smoothed loss, default is 50')
    parser.add_argument('-s', '--snapshots', type=int, default=2000,
        help='how many iters to store the params, default is 1000')
    parser.add_argument('--step-size', type=int, default=2000,
        help='the number of iters to decrease the learning rate, default is 10000')
    parser.add_argument('--display', type=int, default=1,
        help='how many iters display one time, default is 20')
    parser.add_argument('-b', '--balance', type=float, default=1.1,
        help='the parameter to balance the neg and pos, default is 1.1')
    parser.add_argument('-l', '--log', type=str, default='log.txt',
        help='the file to store log, default is log.txt')
    parser.add_argument('-k', type=int, default=1,
        help='the k-th split set of multicue')
    parser.add_argument('--batch-size', type=int, default=1,
        help='batch size of one iteration, default 1')
    parser.add_argument('--crop-size', type=int, default=640,
        help='the size of image to crop, default not crop')
    parser.add_argument('--rate', type=int, default=64,
        help='the size of image to crop, default not crop')
    parser.add_argument('--yita', type=float, default=None,
        help='the param to operate gt, default is data in the config file')
    parser.add_argument('--complete-pretrain', type=str, default=None,
        help='finetune on the complete_pretrain, default None')
```

```
    parser.add_argument('--side-weight', type=float, default=0.5,
        help='the loss weight of sideout, default 0.5')
    parser.add_argument('--fuse-weight', type=float, default=1.1,
        help='the loss weight of fuse, default 1.1')
    parser.add_argument('--gamma', type=float, default=0.1,
        help='the decay of learning rate, default 0.1')
    return parser.parse_args()

if __name__ == '__main__':
    main()
```

```python
# ----------------------------------------
# Matting for data pre- is use for the microscopic images
# ----------------------------------------

import numpy as np
import torch
import torch.optim as optim
import torch.nn as nn
from torch.autograd import Variable
from torch.nn import functional as F
import time
import re
import os
import sys
import cv2
import bdcn
from datasets.dataset import Data
import argparse
import cfg
from matplotlib import pyplot as plt

def sigmoid(x):
    return 1./(1+np.exp(np.array(-1.*x)))


def test(model, args):
    test_root = cfg.config_test[args.dataset]['data_root']
    test_lst = cfg.config_test[args.dataset]['data_lst']
    test_name_lst = test_lst
    # test_name_lst = os.path.join(test_root, 'test_id.txt')
    if 'Multicue' in args.dataset:
        test_lst = test_lst % args.k
        test_name_lst = os.path.join(test_root, 'test%d_id.txt'%args.k)
    mean_bgr = np.array(cfg.config_test[args.dataset]['mean_bgr'])
    test_img = Data(test_root, test_lst, mean_bgr=mean_bgr)
    testloader = torch.utils.data.DataLoader(
        test_img, batch_size=1, shuffle=False, num_workers=8)
    nm = np.loadtxt(test_name_lst, dtype=str)
    print(len(testloader), len(nm))
    assert len(testloader) == len(nm)
    save_res = True
    save_dir = args.res_dir
    if not os.path.exists(save_dir):
        os.mkdir(save_dir)
    if args.cuda:
        model.cuda()
    model.eval()
    data_iter = iter(testloader)
    iter_per_epoch = len(testloader)
    start_time = time.time()
    all_t = 0
    for i, (data, _) in enumerate(testloader):
        if args.cuda:
            data = data.cuda()
        data = Variable(data, volatile=True)
        tm = time.time()
        out = model(data)
        fuse = F.sigmoid(out[-1]).cpu().data.numpy()[0, 0, :, :]
        if not os.path.exists(os.path.join(save_dir, 'fuse')):
            os.mkdir(os.path.join(save_dir, 'fuse'))
        print("result====================",nm[i][0].split("/")[-1])
        cv2.imwrite(os.path.join(save_dir, 'fuse',
        '%s.png'%nm[i][0].split("/")[-1]), fuse*255)
        all_t += time.time() - tm
    print (all_t)
    print ('Overall Time use: ', time.time() - start_time)

def main():
    import time
    print (time.localtime())
    args = parse_args()
```

```
    os.environ['CUDA_VISIBLE_DEVICES'] = args.gpu
    model = bdcn.BDCN()
    model.load_state_dict(torch.load('%s' % (args.model)))
    test(model, args)

def parse_args():
    parser = argparse.ArgumentParser('test BDCN')
    parser.add_argument('-d', '--dataset', type=str, choices=cfg.config_test.keys(),
        default='bsds500', help='The dataset to train')
    parser.add_argument('-c', '--cuda', action='store_true',default=True,
        help='whether use gpu to train network')
    parser.add_argument('-g', '--gpu', type=str, default='0',
        help='the gpu id to train net')
    parser.add_argument('-m', '--model', type=str, default='params/bdcn_final.pth',
        help='the model to test')
    parser.add_argument('--res-dir', type=str, default='result',
        help='the dir to store result')
    parser.add_argument('-k', type=int, default=1,
        help='the k-th split set of multicue')
    return parser.parse_args()

if __name__ == '__main__':
    main()
```

```
# ----------------------------------------
# cropsize to 1024 for data pre- is use for the microscopic images and digital
scanned images
# ----------------------------------------

import cv2
from os.path import join as jn
import os
import math
cropSize = 1024
# computeSize = picsize-cropSize
# sliceTime = 6
# sliceNum = math.ceil(computeSize/(sliceTime-1))
# print(sliceNum)
# exit()
rootPicPath =
"/media/sdc1/medicaldata/皮肤病理新标注2020.10.10/crop/bak/trainValDatacrop640_10x_mer
ge640over"
savePath = "%s_crop1024"%rootPicPath
# savePath = "%s_crop320"%"test"


def getPicList(picPath):
    picList = []
    for root,dirs,files in os.walk(picPath):
        # print(root)
        for file_ in files:
            picList.append((root, file_))
        # if "/deeplab_cls/" in root+"/":
        #     for file_ in files:
        #         picList.append((root,file_))
    return picList

def cropPic(picDir,picName):
    A = cv2.imread(jn(picDir,picName))
    h_,w_,t_ = A.shape
    # print(w_,cropSize,w_/cropSize,int(w_/cropSize))
    slice_i = int(w_/cropSize)+2
    slice_j = int(h_/cropSize)+2
    slice_sizei = int((w_-cropSize)/(slice_i-1))
    slice_sizej = int((h_-cropSize)/(slice_j-1))
    # A = cv2.copyMakeBorder(A, cropSize, cropSize, cropSize, cropSize,
    cv2.BORDER_CONSTANT, value=[0, 0, 0])
    # A = cv2.copyMakeBorder(A, cropSize, cropSize, cropSize, cropSize,
    cv2.BORDER_REFLECT)
    # cv2.imshow("aaa",A)
    # cv2.waitKey()
    savePicPath = jn(picDir.replace(rootPicPath,savePath),picName[:-4])

    if "4倍/" in savePicPath:
        savePicPath = savePicPath.replace("4倍/","/")
    else:
        savePicPath = savePicPath.replace("10倍/","/")
    savePicPath = os.path.dirname(savePicPath)

    if not os.path.exists(savePicPath):
        os.makedirs(savePicPath)
    for i in range(slice_i):
        for j in range(slice_j):

            if i == slice_i-1:
                x0 = w_ - cropSize
            else:
                x0 = i * slice_sizei
            if j == slice_j - 1:
                y0 = h_ - cropSize
            else:
                y0 = j * slice_sizej
            x1 = x0+cropSize
            y1 = y0+cropSize
            A2 = A[y0:y1,x0:x1]
```

```
            # print("------------",y0,y1,x0,x1)




            # print(y0,y1,x0,x1)
            # print(x0,y0,jn(savePicPath,"%d_%d_%s"%(x0,y0,picName)))

            cv2.imwrite(jn(savePicPath,"%d_%d_%d_%d_%s"%(x0,y0,w_,h_,picName)),A2)
            cv2.waitKey(0)


picList = getPicList(rootPicPath)
# exit()


if not os.path.exists(savePath):
    os.mkdir(savePath)
for picPath,picName in picList:
    cropPic(picPath,picName)
    # break
```

```
# ----------------------------------------
# deeplabV3+ segmentation cfg
# ----------------------------------------
import torch
import argparse
import os
import sys
import cv2
import time

class Configuration():
    def __init__(self):
        self.ROOT_DIR =
        os.path.abspath(os.path.join(os.path.dirname("__file__"),'..','..'))
        self.TRAIN_DIR =
        "/media/sdc1/medicaldata/皮肤病理新标注2020.10.10/FromDifferentPathTrainValDat
        a/10x/train"
        # self.TRAIN_DIR = "/media/sdc1/download/HXD/New-Annotation/saveolddata"
        self.EXP_NAME = 'deeplabv3+my'
        self.PRETRAINED = False

        self.DATA_NAME = 'new1020'
        self.DATA_AUG = True
        self.DATA_WORKERS = 0
        self.DATA_RESCALE = 512
        self.DATA_RANDOMCROP = 512
        self.DATA_RANDOMROTATION = 360
        self.DATA_RANDOMSCALE = 0.85
        self.DATA_RANDOM_H = 10
        self.DATA_RANDOM_S = 10
        self.DATA_RANDOM_V = 10
        self.DATA_RANDOMFLIP = 0.5

        self.MODEL_NAME = 'deeplabv3plus'
        self.MODEL_BACKBONE = 'xception'
        self.MODEL_OUTPUT_STRIDE = 16
        self.MODEL_ASPP_OUTDIM = 256
        self.MODEL_SHORTCUT_DIM = 48
        self.MODEL_SHORTCUT_KERNEL = 1
        self.MODEL_NUM_CLASSES = 17
        self.MODEL_SAVE_DIR = os.path.join(self.ROOT_DIR,'model',self.EXP_NAME)

        self.TRAIN_LR = 0.01
        self.TRAIN_LR_GAMMA = 0.1
        self.TRAIN_MOMENTUM = 0.9
        self.TRAIN_WEIGHT_DECAY = 0.00004
        self.TRAIN_BN_MOM = 0.0003
        self.TRAIN_POWER = 0.9
        self.TRAIN_GPUS = 1
        self.TRAIN_BATCHES = 4
        self.TRAIN_SHUFFLE = False
        self.TRAIN_MINEPOCH = 0
        self.TRAIN_EPOCHS = 500
        self.TRAIN_LOSS_LAMBDA = 0
        self.TRAIN_TBLOG = True
        self.TRAIN_CKPT =
        None#os.path.join(self.ROOT_DIR,'model/danetvoc/DANet_xception_VOC2012_epoch60
        _all.pth')
        # self.TRAIN_CKPT =
        "/media/sdc1/model/segmantation/deeplabv3plus-pytorch-master/model/migrate0/de
        eplabv3plus_xception_myData_itr0.pth"#os.path.join(self.ROOT_DIR,'model/danetv
        oc/DANet_xception_VOC2012_epoch60_all.pth')

        self.LOG_DIR = os.path.join(self.ROOT_DIR,'log',self.EXP_NAME)

        self.TEST_MULTISCALE = [1.0]#[0.5, 0.75, 1.0, 1.25, 1.5, 1.75]
        self.TEST_FLIP = False
        # self.TEST_CKPT =
        None#os.path.join(self.ROOT_DIR,'model/danetvoc/DANet_xception_VOC2012_epoch60
        _all.pth')
        self.TEST_GPUS = 1
```

```
        self.TEST_BATCHES = 1
        self.TEST_CKPT =
        "/media/sdc1/model/segmantation/deeplabv3plus-pytorch-master2/model/deeplabv3+
        my10x202010/deeplabv3plus_xception_new1020_itr70000.pth"
        # self.TEST_CKPT =
        "/media/sdc1/model/segmantation/deeplabv3plus-pytorch-master2/model/migrate1_1
        594462287/deeplabv3plus_xception_myData_epoch6000_all.pth"
        # self.TEST_CKPT =
        "/media/sdc1/model/segmantation/deeplabv3plus-pytorch-master2/model/deeplabv3+
        my/deeplabv3plus_xception_myData_epoch10000_all.pth"


        self.__check()
        self.__add_path(os.path.join(self.ROOT_DIR, 'lib'))

    def __check(self):
        if not torch.cuda.is_available():
            raise ValueError('config.py: cuda is not avalable')
        if self.TRAIN_GPUS == 0:
            raise ValueError('config.py: the number of GPU is 0')
        #if self.TRAIN_GPUS != torch.cuda.device_count():
        #   raise ValueError('config.py: GPU number is not matched')
        if not os.path.isdir(self.LOG_DIR):
            os.makedirs(self.LOG_DIR)
        if not os.path.isdir(self.MODEL_SAVE_DIR):
            os.makedirs(self.MODEL_SAVE_DIR)

    def __add_path(self, path):
        if path not in sys.path:
            sys.path.insert(0, path)



cfg = Configuration()
```

```python
# ----------------------------------------
# deeplabV3+ segmentation for different pathological characteristics recognition
# ----------------------------------------

import torch
import torch.nn as nn
import torchvision
import torchvision.transforms as transforms
import os
import sys
import numpy as np

from config import cfg
from datasets.generateData import generate_dataset
from net.generateNet import generate_net
import torch.optim as optim
from PIL import Image
from tensorboardX import SummaryWriter
from torch.utils.data import DataLoader
from net.loss import MaskCrossEntropyLoss, MaskBCELoss, MaskBCEWithLogitsLoss
from net.sync_batchnorm.replicate import patch_replication_callback
from torch.cuda._utils import _get_device_index

def train_net():
    if len(sys.argv)>2:
        cfg.TRAIN_CKPT = sys.argv[1]
        cfg.TRAIN_DIR = sys.argv[2]
    print("ckpt",cfg.TRAIN_CKPT)
    print("traindir",cfg.TRAIN_DIR)
    dataset = generate_dataset(cfg.DATA_NAME, cfg, 'train', cfg.DATA_AUG)
    dataloader = DataLoader(dataset,
                batch_size=cfg.TRAIN_BATCHES,
                shuffle=cfg.TRAIN_SHUFFLE,
                num_workers=cfg.DATA_WORKERS,
                drop_last=True)

    net = generate_net(cfg)
    if cfg.TRAIN_TBLOG:
        from tensorboardX import SummaryWriter
        # Set the Tensorboard logger
        tblogger = SummaryWriter(cfg.LOG_DIR)



    print('Use %d GPU'%cfg.TRAIN_GPUS)
    device = torch.device(0)

    # device_ids = list(range(torch.cuda.device_count()))
    # device_ids = list(map(lambda x: _get_device_index(x, True), device_ids))
    # net.cuda(device_ids[0])
    if cfg.TRAIN_GPUS > 1:
        net = nn.DataParallel(net)
        patch_replication_callback(net)
    net.to(device)


    if cfg.TRAIN_CKPT and cfg.TRAIN_CKPT !="None":
        pretrained_dict = torch.load(cfg.TRAIN_CKPT)
        net_dict = net.state_dict()
        pretrained_dict = {k: v for k, v in pretrained_dict.items() if (k in
        net_dict) and (v.shape==net_dict[k].shape)}
        net_dict.update(pretrained_dict)
        net.load_state_dict(net_dict)
        # net.load_state_dict(torch.load(cfg.TRAIN_CKPT),False)

    criterion = nn.CrossEntropyLoss(ignore_index=255)
    optimizer = optim.SGD(
        params = [
            {'params': get_params(net,key='1x'), 'lr': cfg.TRAIN_LR},
            {'params': get_params(net,key='10x'), 'lr': 10*cfg.TRAIN_LR}
        ],
```

```
        momentum=cfg.TRAIN_MOMENTUM,
        weight_decay=cfg.TRAIN_WEIGHT_DECAY,
    )

    #scheduler = optim.lr_scheduler.MultiStepLR(optimizer,
    milestones=cfg.TRAIN_LR_MST, gamma=cfg.TRAIN_LR_GAMMA, last_epoch=-1)
    itr = cfg.TRAIN_MINEPOCH * len(dataloader)
    max_itr = cfg.TRAIN_EPOCHS*len(dataloader)
    running_loss = 0.0
    tblogger = SummaryWriter(cfg.LOG_DIR)

    for epoch in range(cfg.TRAIN_MINEPOCH, cfg.TRAIN_EPOCHS):
        #scheduler.step()
        #now_lr = scheduler.get_lr()
        for i_batch, sample_batched in enumerate(dataloader):
            now_lr = adjust_lr(optimizer, itr, max_itr)
            inputs_batched, labels_batched = sample_batched['image'],
            sample_batched['segmentation']
            optimizer.zero_grad()
            labels_batched = labels_batched.long().to(0)

            #0foreground_pix =
            (torch.sum(labels_batched!=0).float()+1)/(cfg.DATA_RESCALE**2*cfg.TRAIN_BA
            TCHES)
            # import cv2
            # im_ =
            (inputs_batched.cpu().numpy()*255).astype(np.uint8)[0].transpose(1,2,0)
            # la = (labels_batched.cpu().numpy()*80).astype(np.uint8)[0]
            # print(labels_batched)
            # import time
            # cv2.imwrite(str(time.time())+".png",im_)
            # cv2.imwrite(str(time.time())+".png",la)
            # cv2.waitKey(0)
            # exit()
            inputs_batched = inputs_batched.to(device)
            predicts_batched = net(inputs_batched)
            predicts_batched = predicts_batched.to(0)
            loss = criterion(predicts_batched, labels_batched)

            loss.backward()
            optimizer.step()

            running_loss += loss.item()

            print('epoch:%d/%d\tbatch:%d/%d\titr:%d\tlr:%g\tloss:%g ' %
                (epoch, cfg.TRAIN_EPOCHS, i_batch,
                dataset.__len__()//cfg.TRAIN_BATCHES,
                itr+1, now_lr, running_loss))
            if cfg.TRAIN_TBLOG and itr%100 == 0:
                #inputs =
                np.array((inputs_batched[0]*128+128).numpy().transpose((1,2,0)),dtype=
                np.uint8)
                inputs = inputs_batched.cpu().numpy()[0]
                #inputs = inputs_batched.numpy()[0]/2.0 + 0.5
                labels = labels_batched[0].cpu().numpy()
                labels_color = dataset.label2colormap(labels).transpose((2,0,1))
                predicts = torch.argmax(predicts_batched[0],dim=0).cpu().numpy()
                predicts_color = dataset.label2colormap(predicts).transpose((2,0,1))
                pix_acc = np.sum(labels==predicts)/(cfg.DATA_RESCALE**2)

                tblogger.add_scalar('loss', running_loss, itr)
                tblogger.add_scalar('lr', now_lr, itr)
                tblogger.add_scalar('pixel acc', pix_acc, itr)
                tblogger.add_image('Input', inputs, itr)
                tblogger.add_image('Label', labels_color, itr)
                tblogger.add_image('Output', predicts_color, itr)
            running_loss = 0.0

            if itr % 5000 == 0 and itr:
                save_path =
                os.path.join(cfg.MODEL_SAVE_DIR,'%s_%s_%s_itr%d.pth'%(cfg.MODEL_NAME,c
```

```
                fg.MODEL_BACKBONE,cfg.DATA_NAME,itr))
                torch.save(net.state_dict(), save_path)
                print('%s has been saved'%save_path)

            itr += 1

    save_path =
    os.path.join(cfg.MODEL_SAVE_DIR,'%s_%s_%s_epoch%d_all.pth'%(cfg.MODEL_NAME,cfg.MOD
    EL_BACKBONE,cfg.DATA_NAME,cfg.TRAIN_EPOCHS))
    torch.save(net.state_dict(),save_path)
    if cfg.TRAIN_TBLOG:
        tblogger.close()
    print('%s has been saved'%save_path)

def adjust_lr(optimizer, itr, max_itr):
    now_lr = cfg.TRAIN_LR * (1 - itr/(max_itr+1)) ** cfg.TRAIN_POWER
    optimizer.param_groups[0]['lr'] = now_lr
    optimizer.param_groups[1]['lr'] = 10*now_lr
    return now_lr

def get_params(model, key):
    for m in model.named_modules():
        if key == '1x':
            if 'backbone' in m[0] and isinstance(m[1], nn.Conv2d):
                for p in m[1].parameters():
                    yield p
        elif key == '10x':
            if 'backbone' not in m[0] and isinstance(m[1], nn.Conv2d):
                for p in m[1].parameters():
                    yield p

if __name__ == '__main__':
    train_net()
```

```python
# ----------------------------------------
# efficientNet B1 train process for four subtype classification
# ----------------------------------------

from torchvision import datasets, transforms
import torch
from torch import nn
import torch.optim as optim
import argparse
import warnings
import torch.optim.lr_scheduler as lr_scheduler
from torch.utils.data.dataloader import default_collate
from efficientnet_pytorch import EfficientNet  # EfficientNet lib
from myLabelSmooth import LabelSmoothSoftmaxCE
from myDataset4_10_1116 import MyDataset4_10
import os

os.environ['CUDA_VISIBLE_DEVICES'] = '0'
warnings.filterwarnings("ignore")

data_root_dir =
"/media/sdc1/medicaldata/皮肤病理新标注2020.10.10/trainValDataClassify/分类图片-筛选20
200104copy/train0111"
# num_classes
num_classes = 5
# batch_size
batch_size = 8
# epoch
EPOCH = 201
# feature_extract = True ture for feature extraction, false for Fine-tuning
feature_extract = False
# pre_epoch
pre_epoch = 0  # Define the number of times the data set has been traversed


def my_collate_fn(batch):
    '''
    batch: (data, label)
    '''
    # Data filtered None
    batch = list(filter(lambda x: x[0] is not None, batch))
    if len(batch) == 0: return torch.Tensor()
    return default_collate(batch)  # Use the default method to splice the filtered
    batch data


# input_size = 512
input_size = 640
# EfficientNet use and fine-tune
# net = EfficientNet.from_pretrained('efficientnet-b4')
net = EfficientNet.from_name('efficientnet-b1',in_channels = 40,channel_divide=6)
net._fc.out_features = num_classes
device = torch.device("cuda:0" if torch.cuda.is_available() else "cpu")
if torch.cuda.device_count() > 1:
    net = nn.DataParallel(net)
net.to(device)
# net.load_state_dict(torch.load('./model/net_pre.pth'))
net = net.to(device)
# data_transforms
data_transforms = {
    'train': transforms.Compose([
        transforms.Resize(input_size),
        transforms.CenterCrop(input_size),
        transforms.RandomAffine(degrees=0, translate=(0.05, 0.05)),
        transforms.RandomHorizontalFlip(),
        transforms.ToTensor(),
        transforms.Normalize(mean=[0.485, 0.456, 0.406], std=[0.229, 0.224, 0.225]),

    ]),

    'val': transforms.Compose([
```

```
            transforms.Resize(input_size),
            transforms.CenterCrop(input_size),
            transforms.ToTensor(),
            transforms.Normalize(mean=[0.485, 0.456, 0.406], std=[0.229, 0.224, 0.225])
        ]),
    }
    trainVal = ['train','val']

    #Create training and validation datasets
    image_datasets = {x:
    MyDataset4_10(os.path.join(data_root_dir,"trainValData_person0111", x ,"data","4x"),

                                                   os.path.join(data_root_dir,"trainValData_person0111
                                                   ", x ,"data","10x"),

                                                   os.path.join(data_root_dir,"trainValDataCrop640cls_
                                                   merge640over_person_move0111", x
                                                   ,"deeplab_cls","4x"),

                                                   os.path.join(data_root_dir,"trainValDataCrop640cls_
                                                   merge640over_person_move0111", x
                                                   ,"deeplab_cls","10x"),size_=640) for x in trainVal}

    # Create training and validation dataloaders
    dataloaders_dict = {
        x: torch.utils.data.DataLoader(image_datasets[x], batch_size=batch_size,
        shuffle=True, num_workers=12,
                                                   collate_fn=my_collate_fn) for x in trainVal}
    # b = str(image_datasets[trainVal[0]])
    # c = image_datasets[trainVal[1]].class_to_idx
    # print(b,c)
    # exit()
    parser = argparse.ArgumentParser(description='PyTorch DeepNetwork Training')
    parser.add_argument('--outf', default='./model/model4+10_0111_B1', help='folder to
    output images and model checkpoints')  # Output result save path

    args = parser.parse_args()
    params_to_update = net.parameters()

    print("Params to learn:")
    if feature_extract:
        params_to_update = []
        for name, param in net.named_parameters():
            if param.requires_grad == True:
                params_to_update.append(param)
                print("\t", name)
    else:
        for name, param in net.named_parameters():
            if param.requires_grad == True:
                print("\t", name)


    def main():
        if not os.path.exists(args.outf):
            os.makedirs(args.outf)
        if len(os.listdir(args.outf))>2:
            print("Dir %s have more files than 2"%args.outf)
            exit()
        ii = 0
        LR = 1e-4  # Learning rate
        best_acc = 0  # initial the best test accuracy
        print("Start Training, DeepNetwork!")  # Define the number of times to traverse
        the data set

        # criterion
        criterion = LabelSmoothSoftmaxCE()
        # optimizer
        optimizer = optim.Adam(params_to_update, lr=LR, betas=(0.9, 0.999), eps=1e-9)
        # scheduler
        scheduler = lr_scheduler.ReduceLROnPlateau(optimizer, mode='max', factor=0.7,
        patience=3, verbose=True)
```

```python
    with open("../txt/acc.txt", "w") as f:
        with open("../txt/log.txt", "w")as f2:
            for epoch in range(pre_epoch, EPOCH):
                # scheduler.step(epoch)
                print('\nEpoch: %d' % (epoch + 1))
                net.train()
                sum_loss = 0.0
                correct = 0.0
                total = 0.0

                for i, data in enumerate(dataloaders_dict[trainVal[0]], 0):
                    # input data
                    length = len(dataloaders_dict[trainVal[0]])
                    input, target,_name = data
                    # print(input.shape,target)
                    input, target = input.to(device), target.to(device)
                    # train
                    optimizer.zero_grad()
                    # forward + backward
                    output = net(input)
                    loss = criterion(output, target)
                    loss.backward()
                    optimizer.step()

                    # Print loss and accuracy every training 1 batch
                    sum_loss += loss.item()
                    _, predicted = torch.max(output.data, 1)
                    total += target.size(0)
                    correct += predicted.eq(target.data).cpu().sum()
                    print('[epoch:%d, iter:%d] Loss: %.03f | Acc: %.3f%% '
                          % (epoch + 1, (i + 1 + epoch * length), sum_loss / (i + 1),
                             100. * float(correct) / float(total)))
                    f2.write('%03d  %05d |Loss: %.03f | Acc: %.3f%% '
                             % (epoch + 1, (i + 1 + epoch * length), sum_loss / (i +
                             1),
                                100. * float(correct) / float(total)))
                    f2.write('\n')
                    f2.flush()

                # Test the accuracy after every 10 epochs of training
                if epoch%10 == 0:
                    print("Waiting Test!")
                    with torch.no_grad():
                        correct = 0
                        total = 0
                        for data in dataloaders_dict[trainVal[1]]:
                            net.eval()
                            images, labels,_name = data
                            images, labels = images.to(device), labels.to(device)
                            outputs = net(images)
                            # The category with the highest score (outputs.data's
                            index number)
                            _, predicted = torch.max(outputs.data, 1)
                            total += labels.size(0)
                            correct += (predicted == labels).cpu().sum()
                        print('测试分类准确率为：%.3f%%' % (100. * float(correct) /
                        float(total)))
                        acc = 100. * float(correct) / float(total)
                        scheduler.step(acc)

                        # Write each test result into the acc.txt file in real time
                        if (epoch % 20 == 0):
                            print('Saving model......')
                            torch.save(net.state_dict(), '%s/net_%03d.pth' %
                            (args.outf, epoch + 1))
                            # torch.save(net.state_dict(), '%s/net_%03d.pth' %
                            (args.outf, epoch + 1))
                        f.write("EPOCH=%03d,Accuracy= %.3f%%" % (epoch + 1, acc))
                        f.write('\n')
                        f.flush()
                        # Record the best test classification accuracy rate and
```

```
                        write it into the best_acc.txt file
                        if acc > best_acc:
                            f3 = open("../txt/best_acc.txt", "w")
                            f3.write("EPOCH=%d,best_acc= %.3f%%" % (epoch + 1, acc))
                            f3.close()
                            best_acc = acc
                print("Training Finished, TotalEPOCH=%d" % EPOCH)


if __name__ == "__main__":
    main()
```

```python
# ----------------------------------------
# efficientNet B1 test process for four subtype classification
# ----------------------------------------

import torch
from torch import nn
import warnings
from torch.utils.data.dataloader import default_collate
from efficientnet_pytorch import EfficientNet  # EfficientNet lib
from myDataset4_10ceshi import MyDataset4_10
import os

from sklearn.metrics import confusion_matrix

import re


os.environ['CUDA_VISIBLE_DEVICES'] = '0'
warnings.filterwarnings("ignore")

name2classify = {'正常': 0, '海绵': 1, '苔藓': 2, '银屑': 3}
classify2name = ['正常', '海绵', '苔藓', '银屑']

data_root_dir =
"/media/sdc1/medicaldata/皮肤病理新标注2020.10.10/trainValDataClassify/分类图片-筛选20
200104copy"
# TEST_CKPT =
"/media/sdc1/myCode/charm/model/mutiInputClassify/201116/model/model4+10_1225_B2/net_5
51.pth"
TEST_CKPT =
"/media/sdc1/myCode/charm/model/mutiInputClassify/201116/model/model4+10_0104_B1/net_1
61.pth"

# picDir = "/media/sdc1/download/HXD/New-Annotation/saveolddata/train/pic"
# labelDir = "/media/sdc1/download/HXD/New-Annotation/saveolddata/train/label"
picDir = "/media/sdc1/download/HXD/10X/ceshiClassify/image"
labelDir = "/media/sdc1/download/HXD/10X/ceshiClassify/deeplabv3plus_val_cls"

# num_classes
num_classes = 5
# batch_size
batch_size = 1
# epoch
EPOCH = 1500
# feature_extract = True ture for feature extraction, false for Fine-tuning
feature_extract = False
# pre_epoch
pre_epoch = 0  # Define the number of times the data set has been traversed


def my_collate_fn(batch):
    '''
    batch: (data, label)
    '''
    # Data filtered None
    batch = list(filter(lambda x: x[0] is not None, batch))
    if len(batch) == 0: return torch.Tensor()
    return default_collate(batch)  # Use the default method to splice the filtered
    batch data


input_size = 512
# EfficientNet use and fine-tune
net = EfficientNet.from_name('efficientnet-b1',in_channels = 40,channel_divide=6)
net._fc.out_features = num_classes
device = torch.device("cuda:0" if torch.cuda.is_available() else "cpu")
if torch.cuda.device_count() > 1:
    net = nn.DataParallel(net)

net.to(device)
# net.load_state_dict(torch.load('./model/net_pre.pth'))
```

```
model_dict = torch.load(TEST_CKPT, map_location=device)
net.load_state_dict(model_dict)


net = net.to(device)

trainval = "val"


dataset_ = MyDataset4_10(os.path.join(data_root_dir,"trainValData_person", trainval
,"data","4x"),
                                   os.path.join(data_root_dir,"trainValData_person",
                                   trainval ,"data","10x"),

                                   os.path.join(data_root_dir,"trainValDataCrop640cls_
                                   merge640over_person", trainval
                                   ,"deeplab_cls","4x"),

                                   os.path.join(data_root_dir,"trainValDataCrop640cls_
                                   merge640over_person", trainval
                                   ,"deeplab_cls","10x"))


# dataset_ = MyDataset4_10(  os.path.join(data_dir, trainval+"4" ,"image"),
#                            os.path.join(data_dir, trainval+"10" ,"image"),
#                            os.path.join(data_dir, trainval+"4"
,"deeplabv3plus_val_cls"),
#                            os.path.join(data_dir, trainval+"10"
,"deeplabv3plus_val_cls"),train=False)
dataloader_ = torch.utils.data.DataLoader(dataset_, batch_size=batch_size,
shuffle=True,
                                   collate_fn=my_collate_fn)


def main():

    print("Start Testing, DeepNetwork!")  # Define the number of times to traverse
    the data set

    correct = 0
    total = 0
    preList = []
    truthList = []
    file_ =
    open("result/%s_result0104%s.txt"%(trainval,os.path.basename(data_root_dir)),"w+")
    file_2 =
    open("result/%s_result0104%s_readme.txt"%(trainval,os.path.basename(data_root_dir)
    ),"w+")
    file_2.write("scoreList___person___score___pre___label___name4___name10\n")
    file_2.write(str(name2classify))
    file_2.close()

    for data in dataloader_:
        net.eval()
        imageslist, labels, name_list = data


        for i,images in enumerate(imageslist):
            name_ = name_list[i]
            images, labels = images.to(device), labels.to(device)
            print("sahpeeeeeeeeeeee",images.shape)
            # images = torch.reshape(images, (1, 40, 512, 512))
            outputs = net(images)
            # The category with the highest score (outputs.data's index number)
            # _, predicted = torch.max(outputs.data, 1)
            # print("output",outputs)
            outputs = outputs.data[:,:4]
            # print("output2",outputs)
            sort_ = torch.softmax(outputs,1)
            print("predict1111111111",sort_)
```

```
            score_, predict_ = torch.max(sort_, 1)
            print("predict",predict_)
            personnum = re.findall("^\d+", name_list[i][0].split("_")[4])[0]
            # personnum = re.findall("^\d+", name_list[0].split("_")[4])[0]
            result___ =
            str(sort_.cpu().numpy()[0])+"___"+personnum+"___"+str(score_.cpu().numpy()
            [0])+"___"+
            str(predict_.cpu().numpy()[0])+"___"+str(labels.cpu().numpy()[0])+"___"+na
            me_list[i][0]
            print("result----------------",result___)
            file_.write(result___)
            file_.write('\n')
            file_.flush()


            #
            print("----------------",name_[0]+"__"+str(score_.cpu().numpy()[0])+"__"+s
            tr(predict_.cpu().numpy()[0]))
            total += 1
            preList.append(predict_.cpu().numpy()[0])
            truthList.append(labels.cpu().numpy()[0])
            if predict_[0] == labels[0]:
                correct += 1

            # for i,names in enumerate(classify2name):
            #     print("----------------",i,name_)
            #     if names in name_[0]:
            #
            #         total += 1
            #         if predicted == i:
            #             correct += 1


        # total += labels.size(0)
        # correct += (predicted == labels).cpu().sum()
    file_.close()
    Cm = confusion_matrix(truthList,preList,labels=[0, 1, 2, 3])
    print(Cm)
    print('分类准确率为：%.3f%%' % (100. * float(correct) /
    float(total)),correct,total)
    file_3 =
    open("result/%s_acc0104%s.txt"%(trainval,os.path.basename(data_root_dir)),"w+")
    file_3.write(str(name2classify))
    file_3.write(str(Cm))
    file_3.write('\n分类准确率为：%.3f%%' % (100. * float(correct) /
    float(total))+"_correct"+str(correct)+"_total"+str(total))
    file_3.close()


if __name__ == "__main__":
    main()
```
